# Quality of Artemisinin-Containing Antimalarials in Tanzania's Private Sector—Results from a Nationally Representative Outlet Survey

**DOI:** 10.4269/ajtmh.14-0544

**Published:** 2015-06-03

**Authors:** ACT Consortium Drug Quality Project Team and the IMPACT2 Study Team

**Affiliations:** Departments of Clinical Research, Disease Control, Global Health and Development and Infectious Disease Epidemiology, London School of Hygiene & Tropical Medicine, London, United Kingdom; Division of Parasitic Diseases and Malaria, U.S. Centers for Disease Control and Prevention, Atlanta, Georgia; School of Chemistry and Biochemistry, Georgia Institute of Technology, Atlanta, Georgia; Ifakara Health Institute, Dar es Salaam, Tanzania

## Abstract

Ensuring that artemisinin-containing antimalarials (ACAs) are of good quality is a key component of effective malaria treatment. There are concerns that a high proportion of ACAs are falsified or substandard, though estimates are rarely based on representative data. During a nationally representative survey in Tanzania, ACAs were purchased from private retail drug outlets, and the active pharmaceutical ingredient (API) was measured. All 1,737 ACAs contained the labeled artemisinin derivative, with 4.1% being outside the 85–115% artemisinin API range defined as acceptable quality. World Health Organization (WHO) prequalified drugs had 0.1 times the odds of being poor quality compared with non-prequalified ACAs for the artemisinin component. When partner components of combination therapies were also considered, 12.1% were outside the acceptable API range, and WHO prequalified ACAs had 0.04 times the odds of being poor quality. Although the prevalence of poor quality ACAs was lower than reported elsewhere, the minority of samples found to be substandard is a cause for concern. Improvements in quality could be achieved by increasing the predominance of WHO prequalified products in the market. Continued monitoring of quality standards is essential.

## Introduction

Artemisinin-containing antimalarials† †We use the term artemisinin-containing antimalarials (ACAs) to describe any drug containing an artemisinin derivative (i.e., artesunate, artemether, artemisinin, or dihydroartemisinin) either as a monotherapy or in combination with a partner drug. (ACAs) are acknowledged to be highly efficacious for malaria treatment. Artemisinin-based combination therapies (ACTs) are the recommended first-line treatments for uncomplicated *Plasmodium falciparum* malaria^1^ because artemisinin derivatives alone require at least 7 days of treatment compared with 3 for ACT, and because monotherapy use is thought to contribute to the development of artemisinin resistance. Parenteral artesunate monotherapy is recommended for initial treatment of severe cases followed by a complete course of oral ACT.^2^

Ensuring that ACAs are of good quality is therefore a key component in delivering effective malaria case management, but there are concerns that a high proportion may be of poor quality. Although there is debate about precise terminology, poor quality drugs can be considered to fall into two broad categories. Falsified drugs carry false representations of their source or identity, and often contain none of the stated active ingredient.^3^ Substandard drugs are genuine medicines that do not meet the specifications outlined by an accepted pharmacopeia, and contain sub- or supra-therapeutic doses of the active pharmaceutical ingredient (API).^4^ This may be due to several reasons including poor manufacturing practices and degradation during transport and storage.

Since 2010, falsified and substandard antimalarials have been reported with increasing frequency, especially in the private for-profit sector of malaria-endemic countries.^5^–^9^ However, robust estimates of ACA quality are not available, given the limited data and the tendency to generalize broadly from small-scale, non-representative studies. Analysis of a database of antimalarial quality reports found that 30% were of poor quality, though results varied widely, and less than 5% of the studies used random sampling techniques.^10^ Reports of falsified and substandard antimalarials in the private sector cause alarm as these providers are a common source for treatment of fever, and supply a large proportion of antimalarials.^11^,^12^ For example, in a study of treatment-seeking behavior for children with fever in six African countries, between 17% and 53% caregivers reported attending the private for-profit sector.^13^ Drug quality may be particularly problematic in the private sector, as private retailers are under less scrutiny than the public sector and procurements are usually not state-supported.^14^

Concerns about drug quality are not limited to antimalarials; it has been estimated that up to 15% medicines distributed globally are falsified.^15^ However, poor quality ACAs represent a particularly important public health issue in malaria-endemic countries, as they can result in ineffective treatment of malaria, leading to increased mortality and morbidity, as well as potentially contributing to antimalarial drug resistance because of suboptimal dosing. Antimalarial drug resistance was first documented in southeast Asia in the early 1960s,^16^,^17^ and subsequently in sub-Saharan Africa in the early 1980s.^18^,^19^ Of great concern is the recent documentation of resistance to artemisinin in southeast Asia,^20^–^24^ which could heavily compromise malaria control efforts if it were to spread beyond the Greater Mekong subregion. Therefore, assuring continued quality of ACAs dispensed to malaria patients is of the utmost importance.

As noted above, the majority of studies of antimalarial drug quality in the private sector have used convenience or purposive sampling,^25^–^27^ and as a result should not be generalized to the overall population of drug outlets. Where random sampling has been used, in many cases the sampling frame is not clearly specified or the sample size is insufficient to provide robust estimates of prevalence or associations with risk factors.^7^,^25^–^30^ These previous studies offer valuable preliminary data, and have in some cases served an important purpose in alerting drug regulatory authorities to cases of falsified and substandard products. However, they may substantially over- or underrepresent the true prevalence of poor drug quality on a national level, leading to inappropriate or inadequate policy response. The costs of conducting a nationally representative survey of outlets can be high, but in recent years there has been an increase in the number of such surveys with the primary objective of measuring antimalarial availability, price, and market share.^12^,^31^ We used one such survey in mainland Tanzania in 2010 as a basis for the collection of a representative sample of ACAs to estimate the prevalence of poor quality products in private retail outlets and the associated risk factors.

In Tanzania, an estimated 54% people who sought treatment of fever visited a retail drug outlet in 2012.^32^ At this time, Tanzania had three types of retail drug outlets: Part One Pharmacies (hence forward referred to as pharmacies), which should be run by a pharmacist and can stock most medicines; duka la dawa baridi (DLDB), meaning “medicine shop”, which can officially only stock over-the-counter medicines; and accredited drug dispensing outlets (ADDOs), which are DLDBs that have undergone training and been upgraded, and are allowed to sell over-the-counter products and a select number of prescription-only medications.^33^ ACAs are prescription-only medicines in Tanzania and, in general, only permitted in pharmacies with the exception of the first-line antimalarial, artemether–lumefantrine (Alu), which is allowed in ADDOs. In practice, prescriptions are rarely presented when purchasing prescription-only medicines.^34^ Although not officially permitted, many DLDB also stocked Alu, as the National Malaria Control Programme recognized that these outlets provided an important source of antimalarials for many people.^35^ Oral artemisinin-based monotherapies have been banned, but injectable preparations for treatment of severe malaria are still permitted in pharmacies. General retailers are not officially allowed to stock antimalarials, but occasionally do so.^35^

Several studies have highlighted the presence of poor quality antimalarials in Tanzania,^36^–^38^ including one report of a falsified ACA.^39^ The only previous nationally representative study in 2005 showed that 13.4% sulfadoxine–pyrimethamine (SP) samples were substandard, together with 23.8% quinine samples and 7.5% amodiaquine samples, though no falsified medicines were detected, and all ACAs tested contained the stated amount of active ingredient.^40^ Since 2005, stocking of ACAs in the Tanzanian private sector has substantially increased,^35^ highlighting the importance of rigorous and repeated assessment of ACA quality.^12^

## Methods

### Outlet survey.

A nationally representative outlet survey was conducted in 2010 as part of the Independent Evaluation of the Affordable Medicines Facility–malaria (AMFm), a multi-country antimalarial subsidy program.^12^ The outlet survey covered public, private not-for-profit, and private for-profit antimalarial outlets, but we focus here on the private for-profit sector (pharmacies, DLDB, ADDO, and general retailers) where ACA samples were collected. Data collection took place between September and November 2010 before AMFm was implemented. In total, 48 wards throughout mainland Tanzania's 21 regions‡ ‡Tanzania had 21 regions at the time of the study, though this has subsequently been increased to 25. Results are presented based on the 21 regions at the time of data collection. were randomly selected with probability proportional to population size, stratified by urban/rural location, and every outlet with the potential to sell antimalarials was visited in selected wards. Outlets were identified using official lists, by consulting with district pharmacists and other local leaders and by driving or walking down every street within a ward to locate all outlets. Wards were designated as urban or rural based on National Bureau of Statistics Census classifications. Every pharmacy in the district in which the ward was located was also visited, as pharmacies were relatively few in number but thought to be a major source of antimalarials.

The sample size was determined based on the needs of the independent evaluation (full details available in Tougher and others^12^), which required 305 outlets stocking antimalarials in both urban and rural domains. Estimates of the average numbers of outlets per ward were used to estimate the number of wards required to reach this target, leading to the selection of 9 urban and 39 rural wards.

After verbal informed consent, full interviews were conducted in outlets with an antimalarial in stock at the time of visit or within the previous 3 months, with data collected using personal digital assistants (PDAs). Questions about outlet characteristics were asked using a structured questionnaire, and details about every antimalarial in stock at the time of visit were recorded.

### Collection of ACA samples.

A sample of every ACA present at the time of visit was purchased from every private for-profit outlet that reported stocking any ACAs. For prepackaged ACAs, one packet for each age group was purchased; for ampoules/vials, suppositories, and packets of oral pediatric granules for suspension, at least six units were purchased; and for bottles of syrups and powders for suspensions one bottle was purchased.

Each drug sample was sealed in a waterproof bag with desiccant at the time of purchase. All samples were boxed and stored at ambient temperature for maximum 3 months before being sent to the London School of Hygiene & Tropical Medicine (LSHTM; United Kingdom) by air. At LSHTM, samples were stored in the dark at ambient temperatures (22°C) until and during analysis.

### Laboratory analysis.

We measured the quantity of API present for all artemisinin derivatives. In addition, we measured the API amount for partner drugs in selected ACTs. Because of capacity constraints, it was necessary to limit the partner analysis to the most common partner drugs for each generic artemisinin ingredient: lumefantrine, piperaquine, and mefloquine (used in the ACTs Alu, artemisinin–piperaquine, dihydroartemisinin–piperaquine, artesunate–amodiaquine and artesunate–mefloquine).

The physical dimensions and weight of each antimalarial tablet were recorded prior to processing for analysis, to fully document sample characteristics before preparation. Formulations were analyzed for the amount of each API present using high-performance liquid chromatography with ultraviolet photo-diode array detection (HPLC-UV-PDA). Each tablet was pulverized, and dissolved in solvent depending on the stated API: artesunate, artemether, dihydroartemisinin, amodiaquine, sulfamethoxypyrazine/pyrimethamine, and SP were dissolved in methanol; samples containing lumefantrine (LUM) were dissolved in 10% acetic acid in methanol, mefloquine (MF) in methanol/2.0 N hydrochloric acid (MeOH/2.0 N HCl; v/v) and piperaquine (PIP) in methanol/0.1 M HCl (1:1; v/v). Solvent extracts were sonicated followed by centrifuging, and the supernatant injected into the HPLC column to determine the amount of API present. Syrups, powders, suspensions and suppositories were dissolved in methanol. Injectables where the stated API carrier was coconut oil were also dissolved in methanol, with injectables where the carrier was peanut oil dissolved in isopropanol: hexane (v/v; 16:1).

All samples analyzed at LSHTM were sent to the U.S. Centers for Disease Control and Prevention Laboratories, Atlanta, GA, and the Georgia Institute of Technology, Atlanta, GA, for HPLC confirmatory analysis and mass spectrometry (MS) screening, respectively (both groups were blinded to the LSHTM results). Additional laboratory analysis details are given in Supplemental Information.

### Data analysis.

Data analysis was conducted in Stata version 12 (StataCorp LP, College Station, TX). The primary outcome was “drug quality,” defined as the percentage of the stated amount of API detected in each drug component. Under the World Health Organization (WHO) International Pharmacopoeia, the acceptable range for artemisinin-based antimalarials and their companion drugs is to contain 90.0–110.0% active ingredient stated on the label. Reflecting the typically observed experimental variation in HPLC laboratory testing where less than 10 samples of each ACA formulation were tested, we adopted a wider tolerance band of 85–115% for the purposes of this study. Thus, samples were considered to be of “acceptable quality” in terms of the artemisinin derivative component if these samples contained 85–115% artemisinin derivative API as labeled, and acceptable quality in terms of both the artemisinin derivative and partner drug components if they contained 85–115% API, as labeled for both components.

Results were weighted to reflect the sampling strategy for the outlet survey.^35^ The analysis was corrected for clustering at the district level and urban/rural stratification using Stata SVY commands. Statistical tests were evaluated at *P* ≤ 0.05 level of precision.

The outlet survey included data on several potential risk factors for poor quality medicines, including characteristics of the products and the outlets from which they were purchased. Outlet characteristics included type of outlet (pharmacy, drug store [DLDB or ADDO], general store), and urban or rural location. Product characteristics included generic components, date of manufacture, date of expiry, stated manufacturer, stated country of manufacture, dose form (e.g., suspension, tablet, injectable), whether the drug was WHO prequalified at the time of the survey, whether the drug was a fixed-dose combination, and retail price. “Cheap” drugs were defined as drugs with a price in the lowest quartile of all ACAs sampled, stratified by dosage form. Prices were calculated using adult equivalent treatment doses (AETDs), the amount of a drug needed to completely treat a 60-kg adult.^31^ For example, to calculate the price per AETD of a pediatric package of Alu with six standard tablets (20 mg artemether and 120 mg lumefantrine), the price would be multiplied by four to calculate the cost for an adult equivalent dose of 24 tablets.

The association between poor ACA quality and outlet and product characteristics was examined using regression analysis, taking survey design into account. Unordered categorical variables were tested using the Wald test to estimate the overall statistical significance of categorical variables, for example, dose form. Sensitivity analysis was undertaken using a narrower API range for acceptable quality of 90–110% stated API.

### Ethical approval.

Ethical approval was obtained from the Tanzanian National Institute for Medical Research, the Institutional Review Board of the Ifakara Health Institute, the London School of Hygiene & Tropical Medicine Research Ethics Committee, and from the Institutional Review Board of ICF International.

## Results

### Description of sample.

In total, 3,029 private for-profit retail outlets were visited, of which 512 had an antimalarial in stock on the day of visit (Table 1). From the outlets stocking ACAs, 1,737 ACAs were purchased and analyzed for API content: 55 ACAs were documented in outlets but not analyzed because either it was not possible to purchase the ACA or the purchased ACA could not be linked to the relevant outlet at the time of analysis. By generic class, the most frequently analyzed ACA was artemether: 604 doses were analyzed, of which 504 were stated to be Alu, the first-line antimalarial (Table 2). In all, samples from 177 different stated batches were analyzed.

### Outlet and product characteristics.

Of all outlets with any antimalarials in stock at the time of the visit, nearly all pharmacies reported having at least one member of staff with a health-related qualification (97.6%), compared with only 7.0% general retailers (Table 3). Of all outlets with antimalarials in stock, 21.6% had an ACA, and ACA-stocking outlets were identified in 16 of the 21 regions. Stocking patterns varied substantially across outlet types, with 96.0% pharmacies stocking an ACA, compared with 24.5% drug stores (DLDB or ADDO) and 4.6% general retailers. A WHO prequalified ACT was stocked by 71.0% pharmacies, 11.9% drug stores, and 4.6% general retailers. No drug stores or general retailers stocked artemisinin monotherapies, but 39.8% pharmacies stocked injectable artemisinin monotherapies and 2.1% pharmacies stocked oral artemisinin monotherapy.

Overall, 58.1% ACAs were stocked by drug stores, and 67.7% were from urban areas (Table 4). The majority of products were combination therapies (97.0%), and were in tablet form (86.1%). The most commonly stated regions of manufacture were Asia (45.3%), Africa (27.6%), and Europe (20.7%). Only a quarter of ACAs were WHO prequalified (25.7%). The largest proportions of non-prequalified ACAs were stated to be manufactured in Asia (43.9%) and Africa (36.7%), while a smaller proportion were stated to be manufactured in Europe (18.9%).

Only 2.0% ACAs had expired at the time of purchase. However, 67.4% had expired by the time of analysis because of delays in processing and analyzing the samples. Of these, the majority had expired within 12 months of analysis (43.5%), but a substantial minority had expired more than 1 year before the time of analysis (23.5%). The implications of these delays are discussed below.

### Drug quality based on artemisinin component only.

**Figure 1.**
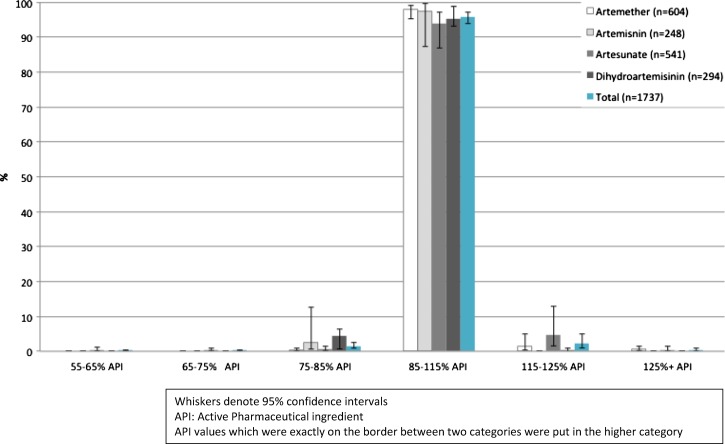
Content of active pharmaceutical ingredient as a percentage of stated content for artemisinin components of all artemisinin-containing antimalarials analyzed.

All drugs analyzed contained the stated artemisinin derivative. Overall 95.9% samples contained between 85% and 115% recommended API for the artemisinin component, with 1.6% containing below 85% and 2.4% containing above 115% (Figure 1). By type of artemisinin derivative, 97.9% artemether and 93.9% artesunate-based drugs were of acceptable quality by this definition. Dihydroartemisinin-based drugs had the highest percentage of products with below 85% stated artemisinin API, while artesunate-containing drugs had the highest percentage of products with more than 115% stated artemisinin API.

The MS analysis conducted at the Georgia Institute of Technology confirmed that all samples contained the stated artemisinin and partner active ingredient, and no other active ingredients were identified. There was a reasonable level of agreement between the HPLC analyses conducted at LSHTM and the U.S. Centers for Disease Control and Prevention (Supplemental Information).

**Figure 2.**
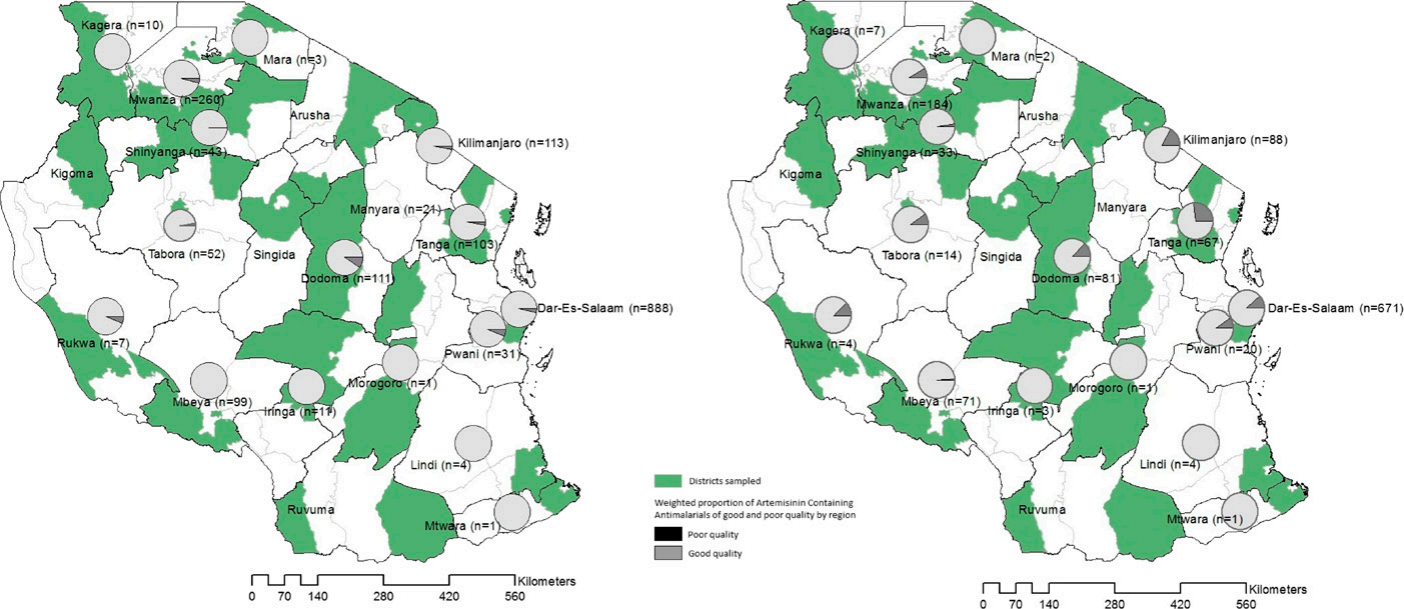
Proportion of (**A**) poor quality artemisinin-containing antimalarials (ACAs) by region based on artemisinin component only, and (**B**) poor quality artemisinin-based combination therapies (ACTs) by region based on artemisinin component and partner drug for selected ACTs (poor quality defined as less than 85% or greater than 115% of stated API).

Figure 2A shows the proportion of poor quality ACAs by region in Tanzania based on the artemisinin component. Poor quality ACAs were purchased from 10 of the 16 regions where ACAs were found. In regions where poor quality ACAs were found, the prevalence ranged from below 1% to 9%.

Table 5 shows the percentage of poor quality drugs based on the artemisinin component by risk factor. On the basis of this bivariate analysis, four risk factors were associated with poor quality at *P* < 0.05: not being WHO prequalified, being relatively expensive (not in lowest price quartile), dose form, and type of artemisinin derivative. Only 0.5% WHO prequalified drugs were of poor quality, compared with 5.4% of those not prequalified. Nearly one-third (29.6%) of pediatric granules were of poor quality, compared with 3.4%, 5.4%, and 3.0% for tablets, suspensions, and injectables, respectively. There was less variation in poor quality by type of artemisinin derivatives: 6.1% artesunate derivatives were of poor quality compared with 4.7%, 2.5%, and 2.1% for dihydroartemisinin, artemisinin, and artemether, respectively. Type of outlet, urban/rural location, whether the product was an ACT rather than a monotherapy, and stated region of manufacture were not found to be associated with poor quality. Expiry at date of analysis was of borderline significance.

Data were too sparse for robust multivariable analysis. However, given the possible association of expiry by date of analysis with quality, we investigated the relationship between drug quality and other risk factors by recalculating their odds ratios (ORs) adjusting for expiry by date of analysis. This adjusted analysis was performed for all other risk factors where *P* < 0.2 in the bivariate analysis (Supplemental Table 1). Adjustment led to no major change in ORs, with the exception of dihydroartemisinin where the OR decreased from 2.3 to 1.9. Only WHO prequalification remained significantly associated with quality after this adjustment (OR 0.1, 95% confidence interval [CI]: 0.01–0.34, *P* = 0.002).

### Drug quality based on artemisinin and partner components.

Laboratory analysis was also conducted on three partner drugs (lumefantrine, piperaquine, and mefloquine) used in the ACTs Alu, artemisinin–piperaquine, dihydroartemisinin–piperaquine, and artesunate–mefloquine. Considering both artemisinin and partner drug quality, 87.9% ACTs were of acceptable quality (Table 6) with 5.1% partner drugs containing higher than the 115% API limit and 3.7% below the 85% API limit. A small proportion of drugs had components that both fell either above (1.6%) or below (0.3%) the 85–115% API range. Poor quality ACTs based on quality of either component were purchased from 10 of the 16 regions where ACTs were found, with prevalence ranging from 1.4% to 26.5%, across regions (Figure 2B).

Table 7 shows the proportion of ACTs that were of poor quality based on both components by risk factor. As with the analysis of artemisinin derivative components alone, not being WHO prequalified, dose form, and type of artemisinin derivative were associated with poor quality based on both components. In addition, expiry at date of analysis was also a risk factor for poor quality when both components were considered. Around one-fifth of artesunate (19.2%)- and dihydroartemisinin (21.1%)-based ACTs were of poor quality. This was nearly double the proportion of poor quality artemisinin-based ACTs (11.6%), and almost seven times that for artemether-based ACTs (3.1%). Price was of borderline significance.

Adjusting for expiry at date of analysis led to no major changes in ORs, with the exception of Europe as stated country of manufacture where the OR decreased from 3.0 to 0.9 (Supplemental Table 2). The only risk factors remaining significant at 0.05 level after adjustment were WHO prequalification (OR 0.04, 95% CI: 0.01–0.2, *P* < 0.001) and type of artemisinin derivative (OR ranged from 5.2 to 7.3, *P* = 0.01).

### Sensitivity analyses.

**Figure 3.**
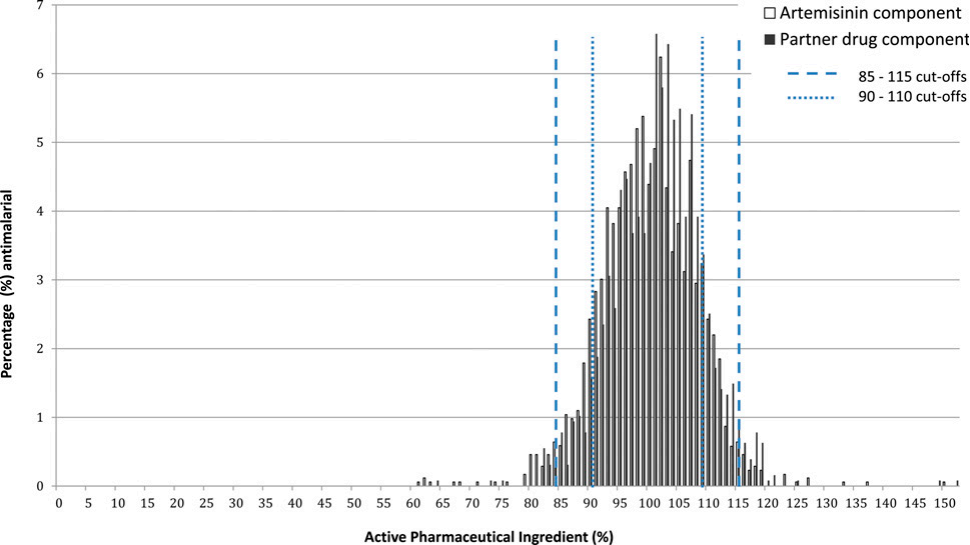
Content of active pharmaceutical ingredient as a percentage of stated content for artemisinin and partner components of all artemisinin-containing antimalarials analyzed, with cutoffs described.

To determine how sensitive results were to the percent API cutoffs used to define poor quality, the analysis was repeated using a narrower range of 90–110%. Using these tighter cutoffs, the percent of products defined as poor quality on the basis of the artemisinin component would have risen from 4.1% to 17.6% and on the basis of both components in ACTs from 12.1% to 30.6% (Figure 3).

## Discussion

This study reports on the quality of artemisinin-containing drugs collected in Tanzania in 2010 as part of a nationally representative survey of private for-profit retail outlets. All samples contained the stated artemisinin derivative, and the majority contained between 85% and 115% stated amount and were therefore considered of acceptable quality. No products contained an incorrect API. However, a substantial minority were substandard: 4.1% for the artemisinin component for all ACAs and 12.1% if both the artemisinin derivative and the partner components were considered for selected ACTs. Dissolution testing was not performed, so the bioavailability of the samples was not measured. The results are not as alarming as might have been expected from previous nonrepresentative surveys. However, drug quality is likely to vary substantially between countries and over time, so one should not assume that these findings can be widely generalized. For example, results from a contemporaneous representative study of malaria-endemic areas in Cambodia also found that all ACAs contained the stated artemisinin derivative, but a higher proportion, 31.6%, were outside the API range of 85–115%.^41^ Studies in Nigeria have found products labeled as ACTs containing no trace of the stated APIs in Enugu and Ilorin states with falsified packaging (Harparkash Kaur, unpublished results), with some products similar to the falsified Coartem^®^ first reported in Angola.^42^

A higher proportion of ACTs were considered poor quality when the quality of the partner drugs was also measured. This highlights the importance of testing both components of ACTs. Moreover, we only had capacity to measure the levels of lumefantrine, mefloquine, and piperaquine, so the quality of other partners, such as SP, amodiaquine, and napthoquine, remains unknown. Partner drug quality is a crucial concern, as it is likely to affect the efficacy of ACTs, given that a 3-day course of an artemisinin alone is insufficient to achieve a high cure rate.^43^ In addition, low levels of the partner drug could leave the artemisinin component unprotected, increasing the likelihood for the development of artemisinin resistance. We have not identified other studies reporting on the quality of partner drugs within ACTs. However, there is substantial evidence of poor quality in commonly used partner drugs such as SP and mefloquine when manufactured as monotherapy products.^39^,^44^

The risk factors associated with a poor quality artemisinin component were not being WHO prequalified, being relatively expensive (not in lowest price quartile), dose form (granules), and type of artemisinin derivative (artesunate). It was notable that drug stores were not found to be more likely than pharmacies to stock poor quality ACAs. There was also no difference between the proportion of poor quality antimalarials found in drug stores in regions where drug stores had been upgraded to ADDOs and those which had yet to be upgraded (data not shown). For partner drugs, risk factors were similar to those for artemisinin components alone. The high proportion of poor quality granular formulation antimalarials were driven by non-prequalified poor quality artesunate–mefloquine, which comprised about half of all artesunate–mefloquine substandard samples.

The sheer volume of drugs sampled in this study and the broad geographic distribution of the outlets from which they were collected limited the speed at which they could be processed for laboratory analysis. In addition, ACAs have a stated short maximum shelf life of 24 months from the date of production. However, despite the high proportion of products expired at analysis, a relatively small minority of these were found to be substandard (5.3% for artemisinin component, 13.5% for both components). Adjusting for expiry at time of analysis did not have a major effect on the magnitude of most ORs. However, after adjustment, only WHO prequalification remained significantly associated with the quality of the artemisinin component only, and only WHO prequalification and type of artemisinin component were associated with quality, when both components of ACTs were considered.

A further methodological limitation is that for each product only one pack of tablets or six units of other formulations were purchased for analysis in the laboratory, compared with the 30 units recommended by USP.^45^ The rationale for this more limited testing was the time constraints involved in chemically analyzing this volume of samples. In addition, purchasing large quantities of ACTs from relatively small medicine stores could have affected access to antimalarials, especially in remote areas. Reflecting this limitation, and recognizing that there is also added variability due to innate variance in HPLC results, we used a relatively broad window for the definition of “acceptable quality” of 85–115% to avoid falsely identifying samples as poor quality. This is not to imply that 85% stated API should be considered an adequate dose, and means that we will have categorized some samples containing inadequate API as “acceptable.” Had a narrower window of 90–110% been used, the percent of products defined as poor quality on the basis of the artemisinin component would have risen from 4.1% to 17.6% and on the basis of both components in ACTs from 12.1% to 30.6%. This high sensitivity of the results to the API cutoffs highlights the need for greater understanding about the therapeutic effects of API variability, and the limitations of testing small numbers of units for each sample.

Weak medicine regulatory systems have been observed in a range of low- and middle-income countries,^46^–^48^ reflecting lack of financial resources, manpower, and capacity.^49^ Capacity to perform laboratory tests on all products entering the market, let alone those available at retail level is therefore inevitably constrained. However, these results indicate that important improvements in quality can be achieved by ensuring that only products meeting WHO prequalification are registered and allowed onto the market. This could lead to concerns that this would cause an increase in prices for consumers. However, data from the Tanzanian survey showed that the median price of WHO prequalified ACAs in tablet form ($5.28 per AETD [interquartile range {IQR} $1.41–$7.04]) was actually lower than the median price of non-WHO prequalified tablet ACAs ($7.93 per AETD [IQR $3.87–$12.67]).

The strength of this study was its large-scale and nationally representative nature, reflecting the opportunity to add sample collection to an ongoing national survey. However, regulatory authorities may not routinely have access to such survey teams. Moreover, the sheer volume of samples collected would be unlikely to be feasible for analysis as part of routine regulatory activities, and even in a research setting leads to long processing times, which would poorly suit a rapid policy response. By contrast, very small-scale convenience samples, collected in response to a perceived threat, frequently lead to alarmist reports overestimating the scale of poor quality products. These reports could themselves have negative consequences, such as undermining user confidence in antimalarials altogether, or encouraging users to revert to less effective drugs.

There is therefore a need to develop “smart” surveillance techniques that provide both robust estimates of drug quality, while being affordable and timely. For example, Newton and others have suggested lot quality assurance sampling (LQAS) of retail outlets as a strategy to provide robust prevalence rates of substandard/falsified drugs over a potentially shorter period.^50^ Quality problems highlighted by LQAS could be further investigated with larger samples to confirm the findings and to identify risk factors. Other strategies to systematically identify high-risk areas for further sampling could include targeting areas where outlet surveys indicate high prevalence of products that are not WHO prequalified or not registered in-country.

## Supplementary Material

Supplemental Information.

## Figures and Tables

**Table 1:** Description of outlets visited and antimalarials obtained

Type of outlets	Outlets enumerated	Outlets meeting screening criteria and interviewed*	Outlets with antimalarials in stock on day of visit	Outlets with artemisinin-containing antimalarials in stock on day of visit	Artemisinin-containing antimalarials audited	Total number of ACAs purchased and analyzed
Pharmacies						
Urban	261	215	214	206	1,601	1,554
Rural	13	8	8	8	54	53
Total	274	223	222	214	1,654	1,607
Drug stores (DLDBs/ADDOs)						
Urban	99	85	85	24	77	73
Rural	172	147	144	30	55	52
Total	271	232	229	54	132	125
General retailers						
Urban	759	1	1	0	0	0
Rural	1,725	80	60	4	6	5
Total	2,484	81	61	4	6	5
Urban total	1,119	301	300	230	1,678	1,627
Rural total	1,910	235	212	42	114	110
Total	3,029	536	512	272	1,792	1,737

ACAs = artemisinin-containing antimalarials; ADDOs = accredited drug dispensing outlets; DLDBs = duka la dawa baridi.

* Outlets were deemed to meet screening criteria if they had an antimalarial in stock or had stocked an antimalarial in the previous 3 months.

**Table 2:** Description of artemisinin-containing antimalarial samples analyzed

Active ingredient	Pharmacy	Drug store (DLDB/ADDO)	General retailer	Total
Artemether				
Artemether monotherapy	100	0	0	100
Alu	463	36	5	504
Total	563	36	5	604
Artemisinin				
Artemisinin monotherapy	0	0	0	0
Artemisinin–napthoquine	121	4	0	125
Artemisinin–piperaquine	160	13	0	173
Total	281	17	0	298
Artesunate				
Artesunate monotherapy	11	0	0	11
Artesunate–amodiaquine	32	5	0	37
Artesunate–mefloquine	295	15	0	310
Artesunate–SP	159	24	0	183
Total	497	44	0	541
Dihydroartemisinin				
Dihydroartemisinin–piperaquine	266	28	0	294
Total	266	28	0	294
Total	1,607	125	5	1,737

ADDOs = accredited drug dispensing outlets; Alu = artemether–lumefantrine; DLDBs = duka la dawa baridi; SP = sulfadoxine–pyrimethamine.

**Table 3:** Characteristics of outlets with antimalarials in stock on the day of visit

Type of outlets	Median number of staff [IQR]	At least one member of staff with health-related qualification* (95% CI)	At least one ACA in stock (95% CI)	At least one ACT in stock (95% CI)	At least one WHO prequalified ACA in stock (95% CI)	At least one artemisinin monotherapy in stock (95% CI)
Pharmacies						
Urban	4 [3, 6]	97.5 (91.3–99.3)	95.8 (90.9–98.1)	95.8 (90.9–98.1)	71.1 (62.3–78.5)	40.2 (29.3–52.2)
Rural	4 [4, 4]	100	100	100	70.1 (32.7–91.9)	50.3 (14.6–85.6)
Total	4 [3, 6]	97.6 (92.3–99.3)	96.0 (91.3–98.2)	96.0 (91.3–98.2)	71.0 (62.5–78.2)	40.6 (30.0–52.2)
Drug stores (DLDBs/ADDOs)						
Urban	2 [1, 2]	96.6 (88.5–99.0)	26.2 (16.4–39.2)	26.2 (16.4–39.2)	13.8 (8.2–22.2)	0
Rural	1 [1, 2]	84.9 (77.5–90.1)	23.8 (12.5–40.7)	23.8 (12.5–40.7)	11.1 (7.5–16.1)	0
Total	2 [1, 2]	88.4 (82.9–92.3)	24.5 (15.7–36.3)	24.5 (15.7–36.3)	11.9 (8.7–16.1)	0
General retailers						
Urban	2 [2, 2]	0	0	0	0	0
Rural	1 [1, 2]	7.1 (2.7–17.0)	4.6 (2.1–9.8)	4.6 (2.1–9.8)	4.6 (2.1–9.8)	0
Total	1 [1, 2]	7.0 (2.7–16.8)	4.6 (2.1–9.8)	4.6 (2.1–9.8)	4.6 (2.1–9.8)	0
Urban total	2 [1, 2]	96.0 (89.8–98.5)	35.6 (25.4–47.5)	35.6 (25.4–47.5)	21.6 (15.8–28.7)	5.6 (3.2–9.7)
Rural total	1 [1, 2]	57.2 (43.2–70.1)	17.1 (9.0–30.1)	17.1 (9.0–30.1)	8.9 (6.2–12.6)	0.1 (0.0–0.4)
Total	1 [1, 2]	66.6 (55.1–76.5)	21.6 (15.0–30.0)	21.6 (15.0–30.0)	12.0 (9.3–15.3)	1.5 (0.8–2.7)

ACA = artemisinin-containing antimalarial; ACT = artemisinin-based combination therapy; ADDOs = accredited drug dispensing outlets; CI = confidence interval; DLDBs = duka la dawa baridi; WHO = World Health Organization.

* A health-related qualification is defined as pharmacy-, nurse-, or medical-doctor-related training. Pharmacy-related training includes studying to a certificate or diploma level. Nurse-related training includes studying nursing to a certificate level (nurse aid) and diploma level. Medical doctor training includes clinical officers who studied medicine to a diploma level and fully qualified physicians.

**Table 4:** Characteristics of artemisinin-containing antimalarial samples analyzed, by generic content

	Artemether (*N* = 604)	Artemisinin (*N* = 248)	Artesunate (*N* = 541)	Dihydroartemisinin (*N* = 294)	All artemisinin-containing antimalarials (*N* = 1,737)	Selected ACTs for which partner drug was also analyzed (*N* = 1,281)
Outlet type						
Pharmacies	42.8 (25.9–61.6)	53.8 (33.0–73.3)	33.4 (16.6–56.0)	34.1 (19.8–51.9)	39.1 (23.6–57.1)	40.9 (25.7–57.9)
Drug stores	48.5 (30.7–66.8)	46.3 (26.7–67.0)	66.6 (44.1–83.4)	65.9 (48.1–80.2)	58.1 (39.9–74.4)	55.2 (37.9–71.3)
General retailers	8.7 (2.4–27.4)	0	0	0	2.8 (0.7–10.5)	4.0 (1.0–14.5)
Location						
Urban	63.2 (43.3–79.4)	86.8 (53.4–97.4)	65.3(28.2–90.0)	66.8 (32.8–89.3)	67.7 (41.8–85.9)	71.8 (52.4–85.4)
Rural	36.8 (20.6–56.7)	13.2 (2.6–46.6)	34.7 (10.0–71.8)	33.2 (10.8–67.2)	32.3 (14.1–58.2)	28.3 (14.6–47.6)
Monotherapy/ACT						
Monotherapy	8.5 (4.6–15.2)	0	0.9 (0.5–1.7)	0	3.0 (1.7–5.2)	0
ACT	91.5 (84.8–95.4)	100	99.1 (98.4–99.5)	100	97.0 (94.9–98.3)	100
Generic name						
Artemether	−	−	−	−	31.7 (23.0–41.9)	41.6 (33.6–50.1)
Artemisinin	−	−	−	−	12.9 (9.2–17.7)	12.5 (9.3–16.4)
Artesunate	−	−	−	−	37.4 (27.9–48.0)	20.0 (15.0–26.0)
Dihydroartemisinin	−	−	−	−	18.1 (14.2–22.7)	26.0 (17.9–36.1)
Dosage form						
Tablet	63.9 (52.1–74.2)	100	93.4 (87.2–96.8)	100	86.1 (80.6–90.3)	84.3 (77.6–89.3)
Suspension	27.6 (18.8–38.5)	0	0	0	8.7 (6.1–12.4)	12.6 (9.5–16.5)
Injectable	8.5 (4.6–15.2)	0	0.7 (0.4–1.4)	0	3.0 (1.7–5.2)	0
Granule	0	0	5.9 (2.6–12.8)	0	2.2 (1.0–5.0)	3.2 (1.4–7.1)
WHO prequalified	61.4 (49.5–72.2)	0	16.8 (7.9–32.2)	0	25.7 (18.7–34.4)	27.7 (19.4–38.0)
Region of stated country of manufacture						
Asia	21.2 (12.5–33.6)	99.8 (99.2–99.9)	15.9 (7.1–31.7)	100	45.3 (41.1–49.5)	49.0 (40.1–58.0)
Africa	31.9 (22.4–43.2)	0.0 (0.0–0.6)	46.8 (27.4–67.1)	0	27.6 (20.4–36.2)	12.4 (9.3–16.4)
Europe	26.6 (20.0–34.5)	0.1 (0.0–0.8)	37.3 (21.7–56.0)	0	20.7 (15.0–27.8)	29.5 (23.3–36.6)
United States	19.3 (7.7–40.6)	0	0.0 (0.0–0.7)	0	6.1 (2.2–16.2)	8.8 (3.2–22.1)
Unknown	1.1 (0.5–2.1)	0	0.0 (0.0–0.4)	0	0.4 (0.2–0.7)	0.3 (0.1–0.7)
Expired at time of purchase	5.3 (2.0–13.3)	0.2 (0.0–1.0)	0.4 (0.0–1.8)	1.2 (0.5–2.7)	2.0 (0.8–5.1)	2.8 (1.1–6.8)
Expired at time of analysis	76.5 (66.2–74.3)	29.3 (18.3–43.4)	57.4 (35.6–76.6)	100	67.4 (56.5–76.8)	83.3 (75.8–88.8)
Price per AETD						
< 25th percentile	39.4 (25.0–56.0)	0.3 (0.0–1.0)	30.2 (7.6–69.4)	2.1 (0.3–14.4)	24.2 (12.6–41.4)	18.2 (10.3–30.0)

ACTs = artemisinin-based combination therapies; AETD = adult equivalent treatment dose; DHA = dihydroartemisinin; WHO = World Health Organization.

**Table 5:** Percentage of artemisinin-containing antimalarial samples that were poor quality by risk factor, and ORs of being poor quality based on artemisinin component only

	*N*	Number of poor quality	Percentage poor quality	Unadjusted OR	*P* value
Outlet type					0.3
Pharmacies	1,601	85	5.7 (4.6–7.1)	1	
Drug stores	124	4	3.2 (1.3–7.9)	0.6 (0.2–1.6)	
General retailers	5	0	0	−	
Location					0.2
Urban	1,621	85	5.0 (3.4–7.4)	1	
Rural	109	4	2.3 (0.8–6.8)	0.5 (0.1–1.5)	
Monotherapy/ACT					0.5
Monotherapy	1,620	86	2.9 (1.2–6.7)	1	
ACT	110	3	4.1 (2.7–6.3)	1.4 (0.5–4.1)	
Generic type					0.02
Artemether	599	16	2.1 (0.9–4.8)	1	
Artemisinin	298	4	2.5 (0.5–12.7)	1.2 (0.2–9.3)	
Artesunate	539	46	6.1 (2.7–13.1)	3.1 (0.7–12.4)	
Dihydroartemisinin	294	23	4.7 (2.6–8.4)	2.3 (1.4–4.0)	
WHO prequalified					0.002
No	1,442	85	5.4 (3.4–8.5)	1	
Yes	308	4	0.5 (0.1–1.9)	0.1 (0.02–0.4)	
Dose form					0.02
Tablet	1,385	55	3.4 (1.8–6.3)	1	
Suspension	192	11	5.4 (1.7–15.8)	1.6 (0.4–7.3)	
Injectable	107	3	3.0 (1.3–6.9)	0.9 (0.3–2.8)	
Granule	46	20	29.6 (9.9–61.5)	10.6 (1.7–65.9)	
Stated region of manufacture					0.1
Asia	850	32	3.0 (1.8–5.1)	1	
Africa	435	15	2.3 (0.9–5.4)	0.8 (0.4–1.6)	
Europe	412	41	10.2 (4.3–22.1)	3.6 (1.0–13.3)	
United States	17	1	0.01 (0.0–5.0)	0.2 (0.02–1.7)	
Expired at date of purchase					–
Not expired	1,703	89	4.2 (2.8–6.3)	1	
Expired	26	0	0	Omitted	
Expired at date of analysis					0.05
Not expired	644	10	1.8 (0.7–4.8)	1	
Expired	1,083	79	5.2 (3.5–7.8)	3.0 (1–8.7)	
Price per AETD					0.03
< 25th percentile	133	9	1.5 (0.6–3.9)	1	
≥ 25th percentile	1,599	80	4.9 (3.3–7.2)	3.3 (1.1–9.8)	

ACT = artemisinin-based combination therapy; AETD = adult equivalent treatment dose; WHO = World Health Organization.

Poor quality defined as less than 85% or greater than 115% of stated API.

**Table 6:** Percentage of selected ACTs by quantity of stated API for artemisinin component and partner drug

(a) All ACTs analyzed (*N* = 1,281)								
Partner drug								
Artemisinin component	%API	55–65	65–75	75–85	85–115	115–125	> 125	Total
	55–65	< 0.1	0	0.1	< 0.1	< 0.1	0	0.2
	65–75	0	0.1	0	< 0.1	0	0	0.2
	75–85	0	0	< 0.1	1.7	0.1	0	1.9
	85–115	0	0	3.3	**87.9**	2.7	0.6	94.4
	115–125	0	< 0.1	0	1.4	1.5	0	2.9
	> 125	0	0	0	0.2	0.1	0	0.3
	Total	< 0.1	0.2	3.4	91.3	4.5	0.6	100
(b) Alu (*N* = 504)								
Lumefantrine								
Artemether drugs	%API	55–65	65–75	75–85	85–115	115–125	> 125	Total
	55–65	0	0	0	0	0	0	0.0
	65–75	0	0	0	0	0	0	0.0
	75–85	0	0	0	< 0.1	0	0	< 0.1
	85–115	0	0	0.2	**96.9**	1.0	0	98.0
	115–125	0	0	0	1.4	0	0	1.4
	> 125	0	0	0	0.6	0	0	0.6
	Total	0.0	0.0	0.2	98.9	1.0	0.0	100
(c) Artemisinin–piperaquine (*N* = 173)								
Piperaquine								
Artemisinin	%API	55–65	65–75	75–85	85–115	115–125	> 125	Total
	55–65	0	0	0	0	0	0	0.0
	65–75	0	0	0	0	0	0	0.0
	75–85	0	0	0	3.6	0	0	3.6
	85–115	0	0	0.9	**88.5**	2.7	4.3	96.4
	115–125	0	0	0	0	0	0	0.0
	> 125	0	0	0	0	0	0	0.0
	Total	0.0	0.0	0.9	92.1	2.7	4.3	100
(d) Artesunate–mefloquine (*N* = 310)								
Mefloquine								
Artesunate	%API	55–65	65–75	75–85	85–115	115–125	> 125	Total
	55–65	<0.1	0	0.6	<0.1	0.2	0	1.0
	65–75	0	0.6	0	0.4	0	0	1.0
	75–85	0	0	0	0.9	0.6	0	1.5
	85–115	0	0	0.3	**80.8**	3.7	< 0.1	84.9
	115–125	0	0.2	0	3.4	7.5	0	11.2
	> 125	0	0	0	0	0.6	0	0.6
	Total	< 0.1	0.8	0.9	85.5	12.6	< 0.1	100
(e) Dihydroartemisinin–piperaquine (*N* = 294)								
Piperaquine								
Dihydroartemisinin	%API	55–65	65–75	75–85	85–115	115–125	> 125	Total
	55–65	0	0	0	0	0	0	0.0
	65–75	0	0	0	0	0	0	0.0
	75–85	0	0	0.2	4.1	0	0	4.2
	85–115	0	0	11.6	**78.9**	4.7	0.1	95.3
	115–125	0	0	0	0.4	0	0	0.4
	> 125	0	0	0	0	0	0	0.0
	Total	0.0	0.0	11.8	83.4	4.7	0.1	100

ACT = artemisinin-based combination therapy; Alu = artemether–lumefantrine; API = active pharmaceutical ingredient.

The percentage of samples containing acceptable quantities of API for both the artemisinin component and partner drug is shown in bold.

**Table 7:** Percentage of ACT samples that were poor quality by risk factor, and ORs of being poor quality based on both artemisinin and partner components for selected ACTs

	*N*	Number of poor quality	Percentage poor quality	Unadjusted OR	*P* value
Outlet type					0.4
Pharmacies	1,180	124	11.0 (9.6–12.6)	1	
Drug stores	91	11	13.9 (7.4–24.5)	1.3 (0.6–2.6)	
General retailers	5	0	0	−	
Location					0.6
Urban	1,202	126	11.2 (8.3–15.0)	1	
Rural	74	9	14.6 (4.7–36.8)	1.35 (0.38–4.84)	
Generic type					0.04
Artemether	499	17	3.1 (1.3–7.5)	1	
Artemisinin	173	14	11.6 (5.3–23.5)	4.0 (1.3–12.9)	
Artesunate	310	65	19.2 (11.6–30.1)	7.3 (2.0–26.6)	
Dihydroartemisinin	294	39	21.1 (11.1–36.4)	8.3 (2.6–25.9)	
WHO prequalified					< 0.001
No	1,015	130	16.4 (11.3–23.2)	1	
Yes	261	5	0.8 (0.3–2.5)	0.04 (0.01–0.1)	
Dose form					0.04
Tablet	1,040	99	11.8 (7.1–18.9)	1	
Suspension	192	12	8.6 (2.9–22.6)	0.7 (0.2–2.6)	
Injectable	0	0	−	−	
Granule	44	24	35.1 (10.1–72.1)	3.9 (0.7–21.9)	
Stated region of manufacture					0.05
Asia	657	56	14.5 (7.9–25.2)	1	
Africa	186	12	8.7 (2.9–22.9)	1.5 (0.6–4.0)	
Europe	404	66	13.2 (7.2–23.1)	3.0 (0.9–10.1)	
United States	17	1	0.6 (0.0–5.1)	0.1 (0.01–1.5)	
Expired at date of purchase					0.2
Not expired	1,257	134	12.3 (8.1–18.2)	1	
Expired	18	1	3.0 (0.3–22.4)	10.2 (0.02–2.1)	
Expired at date of analysis					0.02
Not expired	303	15	5.7 (2.8–11.3)	1	
Expired	972	120	13.5 (8.8–20.2)	2.57 (1.20–5.5)	
Price per AETD					0.05
< 25th percentile	70	8	4.5 (1.3–15.0)	1	
≥ 25th percentile	1,206	127	13.7 (9.4–16.7)	3.36 (0.9–11.6)	

ACT = artemisinin-based combination therapies; AETD = adult equivalent treatment dose. OR = odds ratio; WHO = World Health Organization.

Poor quality defined as less than 85% or greater than 115% of stated API.
