# Supplementary material for: Quality of Artemisinin-Containing Antimalarials in Tanzania's Private Sector—Results from a Nationally Representative Outlet Survey
Source: Am J Trop Med Hyg. 2015 Jun 3;92(Suppl 6):75–86. doi: 10.4269/ajtmh.14-0544 (PMC4455080; doi:10.4269/ajtmh.14-0544)
Supplement: Supplementary file 1 [file SD4.pdf]

SUPPLEMENTAL INFORMATION

DETAILS OF LABORATORY ANALYSIS

High-performance liquid chromatography (HPLC) analysis at the London School of Hygiene & Tropical Medicine (LSHTM; United Kingdom) was conducted using a Dionex Ultimate 3000 system (Thermofisher, Hemel Hempstead, United Kingdom) and separation achieved using a GENESIS AQ 4-μm column (150 × 4.6 mm; Grace Materials Technologies, Cranforth, United Kingdom). The mobile phase was a gradient of ammonium formate (10 mM, pH 2.7) and acetonitrile (v/v; 60:40 to 85:15 over 7.0 minutes). A photo-diode array unit (ultraviolet [UV]-PDA; DAD 3000) was set at 204 nm for the artemisinin derivatives (artesunate, artemether, dihydroartemisinin [DHA]), 360 nm for piperaquine, amodiaquine and lumefantrine and 259 nm for MF. In all cases, the flow rate used was 1.0 mL/min. Calibration curves of each compound were generated by Thermofisher Scientific Dionex Chromeleon 7.2 chromatography data system (CDS) software using known amounts of the corresponding chemical standards. Reference standards of artemisinin, artesunate, artemether, DHA, amodiaquine dichlorodihydrate, and pyrimethamine were purchased from Sigma Aldrich, United Kingdom. Lumefantrine was purchased from World Health Organization (WHO), Switzerland. Mefloquine and sulfadoxine (SP) were a gift from Roche, Basel, Switzerland, and sulfamethoxypyrazine was a gift from Dafra PHARMA, Belgium. Results were expressed as a percentage of the stated amounts of API on the package.

Samples analyzed at LSHTM were sent without the original packaging to two collaborative laboratories at the Georgia Institute of Technology (GT), Atlanta, GA, for MS screening of all samples and the U.S. Centers for Disease Control and Prevention (CDC) Laboratories Atlanta, GA for HPLC confirmatory analysis on a subsample of 14% samples.

HPLC analysis methodology at CDC. In brief, all tablets were pulverized, dissolved in the appropriate solvent and filtered using a 0.22 μm nylon membrane. A portion of the extract was injected into the high-performance liquid chromatographic (HPLC) system. Component separation was achieved using a 150 × 4.6 mm octadecyl silica column and a mobile phase consisting of various proportions of acetonitrile and 0.05 M perchlorate buffer adjusted to a pH of 2.5 with UV detection.

The Bland–Altman plot in Supplemental Figure 1 illustrates the between-laboratory differences of drug samples analyzed by CDC and LSHTM. The mean difference between laboratory results (bias) is 0.15 %API (95% confidence interval [CI]: −0.75 to 1.0) with no significant difference from zero. The 95% CI for the limits of agreement is −19.6 to 19.9 %API for all active ingredients analyzed by both laboratories. Considering that the exact same tablets could not be analyzed by both laboratories, the different HPLC methods used and considering intertablet variability, this was considered a reasonable level of agreement.

MS analysis at GT. MS analyses of drug samples were performed using a direct analysis in real time (DART) ion source coupled to a time-of-flight mass spectrometer (TOF MS). A commercial DART-100 ion source (IonSense, Saugus, MA) was used in-line with a Bruker micrOTOF-Q I mass spectrometer (Bremen, Germany). Detailed description of the DART-100 ion source is available elsewhere.[49,50]

The DART ion source ceramic nozzle was positioned 1 cm away and in-line with the MS inlet. The DART working gas (high purity He, 99.995% Airgas, Atlanta, GA) was supplied to the ion source at a flow rate of 1 L/min and was heated to 250°C. The DART ion source was operated at a needle electrode voltage of −4,000 V, a discharge grid voltage of +200 V, and exit grid voltage of +200 V. These conditions were found to provide maximum sensitivity and good chemical detectability for the various species investigated. To prevent gas overload on the MS vacuum system from the gas flow exiting the ion source, a Vapur interface (IonSense) was used. The MS voltage settings were optimized for maximum ion transmission of 50–1,200 Da with end plate offset −500 V, capillary −2,000 V, dry gas (nitrogen) 1.2 L/min, capillary 200°C, spectra acquired at 1 Hz in the 50–1,200 m/z range in positive ion detection mode.

The instrument was mass calibrated using a 10 μM methanol solution of polyethylene glycol (PEG) 400, PEG 600, and PEG 1000 calibration standards. During calibration, continuous supply of the calibration standard solution for ionization by DART was maintained by delivering the standard solution through a 10-μm i.d. silica capillary in front of the ion source at a flow rate of 3 μL/min using a KD Scientific syringe pump (New Hope, PA). The mass spectrometer provided a mass resolution of ~12,000 at m/z 393.2095 and ~9,000 at m/z 151.0964, and a typical mass accuracy of 2–5 ppm was obtained for acetaminophen as test compound.

All experiments were performed in positive ion detection mode and all samples were analyzed under identical instrumental conditions. For MS analysis of pharmaceuticals in tablet form, the coating was scratched by a razor blade, a few tablet particles were obtained by scratching the inside and outside surfaces of the tablet, the particles were placed on a Kimwipe (Kimberly-Clark Corporation, Dallas, TX), and deposited on the tip of a glass capillary by rubbing it against the Kimwipe. Any liquid injectables were deposited on the capillary by dipping the tip of the glass capillary in the liquid sample vial. The capillary tip with the deposited sample was then introduced in front of the plasma plume exiting the DART source for sample ionization and subsequent detection by mass spectrometry.

The Bruker Daltonics DataAnalysis version 4.0 software package was used for processing of all acquired data. Presence and identity of active pharmaceutical ingredient(s) (API or APIs) in samples were established by matching m/z values measured by the mass spectrometer with the calculated exact m/z values (within a maximum uncertainty of 0.005 Da) of the API, together with the isotopic abundances, and adduct, or fragment ions listed in an in-house built database.

The MS analysis confirmed that all samples contained the stated artemisinin and partner active ingredient, and no other active ingredients were identified.

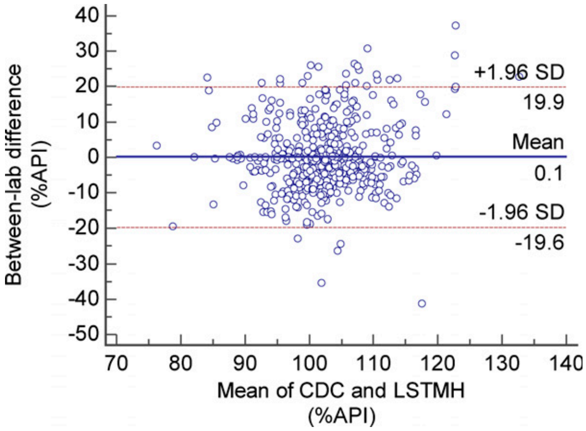

Supplemental Figure 1. Bland–Altman plot of inter laboratory comparison of percent active pharmaceutical ingredients (%APIs) determined from U.S. Centers for Disease Control and Prevention (CDC) and London School of Hygiene and Tropical Medicine (LSHTM) laboratories. Data include the analysis of artemether, artesunate, dihydroartemisinin, lumefantrine, mefloquine, and piperaquine ($N = 488$).

Supplemental Table 1

Adjusted ORs of being poor quality based on artemisinin component only by risk factors where $P < 0.2$ in bivariate analysis, controlling for whether drugs were expired at time of analysis

	N	Number of poor quality	Adjusted OR*	*P* value
Generic type				0.01
Artemether	499	17	1	
Artemisinin	173	14	5.2 (1.2–22.9)	
Artesunate	310	65	7.0 (1.9–25.7)	
Dihydroartemisinin	294	39	7.3 (2.3–23.5)	
WHO prequalified				< 0.001
No	1,015	130	1	
Yes	256	5	0.04 (0.01–0.2)	
Dose form				0.1
Tablet	1,040	99	1	
Suspension	192	12	0.6 (0.2–2.3)	
Injectable	0	0	–	
Granule	44	24	3.4 (0.6–19.1)	
Stated region of manufacture				0.05
Asia	657	56	1	
Africa	186	12	0.5 (0.1–1.7)	
Europe	404	66	0.9 (0.3–2.5)	
United States	17	1	0.03 (1.2–6.2)	
Price per AETD				0.06
< 25th percentile	70	8	1	
≥ 25th percentile	1,206	127	3.3 (0.7–11.4)	

ACTs = artemisinin-based combination therapies; AETD = adult equivalent treatment dose; OR = odds ratio; WHO = World Health Organization.

Poor quality defined as less than 85% or greater than 115% of stated active pharmaceutical ingredient (API).

*After controlling for whether expired at time of analysis.

Supplemental Table 2

Adjusted ORs of being poor quality based on both artemisinin and partner components for selected ACTs by risk factors where $P < 0.2$ in bivariate analysis, controlling for whether drugs were expired at time of analysis

	N	Number of poor quality	Adjusted OR*	*P* value
Generic type				0.2
Artemether	599	16	1	
Artemisinin	298	4	2.1 (0.3–14.2)	
Artesunate	539	46	3.6 (1.0–13.4)	
Dihydroartemisinin	294	23	1.9 (1.1–3.4)	
WHO prequalified				0.002
No	1,442	85	1	
Yes	308	4	0.1 (0.01–0.34)	
Dose form				0.1
Tablet	1,385	55	1	
Suspension	192	11	1.3 (0.3–5.6)	
Injectable	107	3	1.3 (0.4–4.8)	
Granule	46	20	8.3 (1.3–54.8)	
Stated region of manufacture				0.1
Asia	850	32	1	
Africa	435	15	0.9 (0.4–2.2)	
Europe	412	41	3.6 (1.0–13.1)	
United States	17	1	0.2 (0.02–1.7)	
Price per AETD				0.06
< 25th percentile	133	9	1	
≥ 25th percentile	1,599	80	2.9 (0.9–8.4)	

ACTs = artemisinin-based combination therapies; AETD = adult equivalent treatment dose; OR = odds ratio; WHO = World Health Organization.

Poor quality defined as less than 85% or greater than 115% of stated active pharmaceutical ingredient (API).

*After controlling for whether expired at time of analysis.
